# A Comprehensive Review on Zeolite Chemistry for Catalytic Conversion of Biomass/Waste into Green Fuels

**DOI:** 10.3390/molecules27238578

**Published:** 2022-12-05

**Authors:** Umair Yaqub Qazi, Rahat Javaid, Amir Ikhlaq, Asif Hussain Khoja, Faisal Saleem

**Affiliations:** 1 Department of Chemistry, College of Science, University of Hafr Al Batin, P.O. Box 1803, Hafr Al Batin 39524, Saudi Arabia; 2 Renewable Energy Research Center, Fukushima Renewable Energy Institute, National Institute of Advanced Industrial Science and Technology, AIST, 2-2-9 Machiikedai, Koriyama 963-0298, Fukushima, Japan; 3 Institute of Environmental Engineering and Research, University of Engineering and Technology, Lahore 54890, Pakistan; 4 Fossil Fuels Laboratory, Department of Thermal Energy Engineering, U.S.-Pakistan Centre for Advanced Studies in Energy (USPCAS-E), National University of Sciences & Technology (NUST), Sector H-12, Islamabad 44000, Pakistan; 5 Department of Chemical and Polymer Engineering, University of Engineering and Technology, Faisalabad Campus, Lahore 38000, Pakistan

**Keywords:** zeolites, biomass, acidic catalysis, biomass conversion, platform chemicals

## Abstract

Numerous attempts have been made to produce new materials and technology for renewable energy and environmental improvements in response to global sustainable solutions stemming from fast industrial expansion and population growth. Zeolites are a group of crystalline materials having molecularly ordered micropore arrangements. Over the past few years, progress in zeolites has been observed in transforming biomass and waste into fuels. To ensure effective transition of fossil energy carriers into chemicals and fuels, zeolite catalysts play a key role; however, their function in biomass usage is more obscure. Herein, the effectiveness of zeolites has been discussed in the context of biomass transformation into valuable products. Established zeolites emphasise conversion of lignocellulosic materials into green fuels. Lewis acidic zeolites employ transition of carbohydrates into significant chemical production. Zeolites utilise several procedures, such as catalytic pyrolysis, hydrothermal liquefaction, and hydro-pyrolysis, to convert biomass and lignocelluloses. Zeolites exhibit distinctive features and encounter significant obstacles, such as mesoporosity, pore interconnectivity, and stability of zeolites in the liquid phase. In order to complete these transformations successfully, it is necessary to have a thorough understanding of the chemistry of zeolites. Hence, further examination of the technical difficulties associated with catalytic transformation in zeolites will be required. This review article highlights the reaction pathways for biomass conversion using zeolites, their challenges, and their potential utilisation. Future recommendations for zeolite-based biomass conversion are also presented.

## 1. Introduction

**Figure 1:**
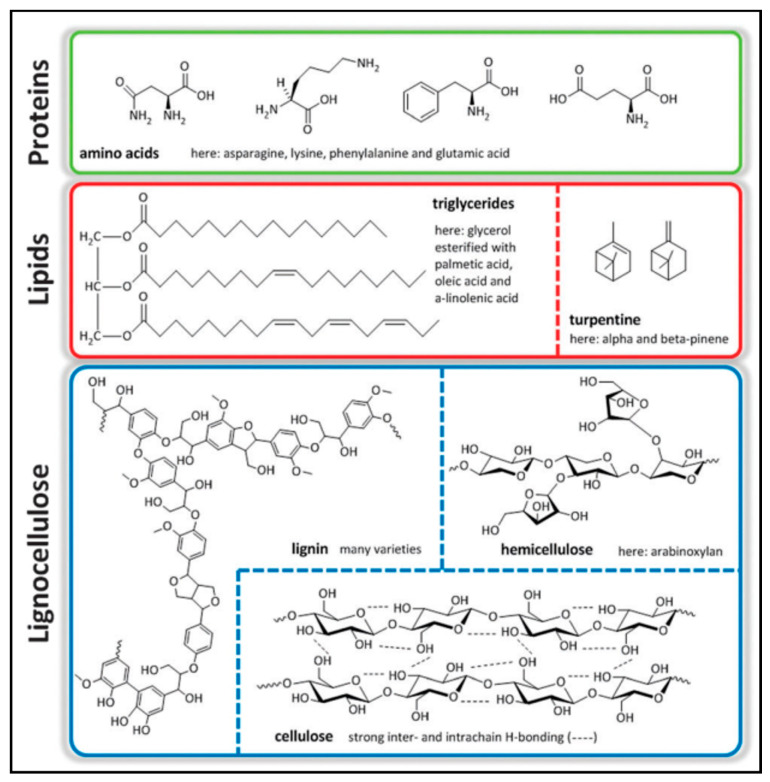
Illustrates the essential components of biomass feedstock [10] (Open Access).

Biomass transformation into chemicals and fuel with innovative catalytic trajectories emergence has become prominent for industries in recent times. Objectives such as the variances and the viability of raw materials instigate that biomass plays a critical role in favour of a sustainably run society by using automation in conversion. Biomass was also the primary energy source at the traditional level before the industrial age. However, it has recently shared a small proportion of the total global power requirement. For this, a method such as thermal combustion is employed in underprivileged nations. Biomass utilised for chemical and fuel production with existing industrial operations is at a nascent stage. The essential biomass sources include lignocellulose and lipids comprising triglycerides from animal fats, vegetable fats, microalgae, and turpentine [1]. The most prevalent and abundant of the three is lignocellulose, which is made of cellulose, hemicellulose, and lignin [2,3,4,5,6,7,8,9]. Finally, a smaller subset of protein-based fractions is derived from either animal or vegetable sources. Figure 1 depicts the most significant biomass feedstock components [10]. Biomass leftovers and dumpsters are produced as by-products when the intended raw goods are grown, processed, and utilised in contrast to biomass specifically farmed for energy purposes [11]. In-depth biomass leftovers are divided into primary, secondary, and tertiary categories. Primary leftovers, including certain maise stalks, stems, leaflets, and straw, are frequently formed during field planting of specific farming and forest products. On the other hand, secondary leftovers are created when agricultural products are transformed into finished goods. Agricultural and industrial wastes, including firewood, coffee husks, corn husk, sugarcane bagasse, and palm kernel cake, are examples of secondary leftovers. Tertiary leftovers, however, emerge after humans and other living creatures have eaten a biomass-derived commodity. These residues may exist in municipal solid waste before transforming into sewage sludge [12,13].

Lange et al. (2012) have stated a prominent illustration of xylose to furfural transformation based on the conventional bio-derived procedure. Furfural could be used as a herbicide and lubricant [14]. Luo (2020) stated that the biomass pyrolysis process in the presence of a catalyst leads to fast conversion of aromatics compounds toluene, xylenes, benzenes, olefins, etc. [15]. During recent decades, catalysis emerged as a technical field under guidance of installed fuels and chemical industries. Catalytic chemistry’s immense understanding is related to existing industrial operations and emphasises improvement in non-functionalised, less reactive, and polar hydrocarbons [11]. To facilitate these hydrocarbons into high-grade fuels, activation of carbon–carbon and carbon–hydrogen bonds is needed with several procedures. Consider hydrocarbon cracking and isomerisation methods. Hydrocarbon cracking (hydrocracking and fluid catalytic cracking) involves transformation of the boiling point of hydrocarbon. Isomerisation leads to up-gradation in octane numbers [16,17,18,19,20,21]. Both methods significantly utilise zeolite catalysts. Serrano et al. (2018) displayed significant variations in fossil fuels consisting of carbon and hydrogen [22].

Several industrial processes, such as petroleum refining, led to direct formation of aromatic hydrocarbons, and the thermal cracking procedure of naphtha results in olefins formation as well. It could be intriguing to emphasise the fossil gases instead of the thermal cracking approach of naphtha for aromatic compound formation. Therefore, shale gas (natural fossil gas) conversion into aromatic compounds could be targeted in the foreseeable future, as stated by Cheng, Jae (2012) [23]. In China, syngas and inexpensive coal were found to be associated with zeolites-based transformation into olefins. A substantial fundamental difficulty in catalysis has been observed owing to modern synthetic techniques employing biomass as its primary source. Oil can be categorised into homogeneous material with significant elements, illustrated by non-functional groups, less reactiveness, and a polar hydrocarbon particle based on a chemical standpoint. In contrast, biomass’s chemical structure and composition rely heavily on the resource. Biomass is constituted of highly reactive materials and expensive functional and polar groups. For effective transformation of initial material into products such as fuels and chemicals, modern chemo-catalytic passages need to be designed [24,25,26,27,28]. Zeolites are crystalline micropore aluminosilicates with favourable physicochemical properties, such as a large surface area and sub-nanometre-scale three-dimensional space apertures. The three-dimensional system offers hydrothermal and significant stability at a chemical level and shape-selectivity features to zeolites. SiO_4_ and AlO_4_ tetrahedral combine to form a crystalline lattice (three-dimensional microporous structure of zeolites). In general, zeolites’ electroneutrality is retained by cationic charges that offset [AlO_4_] tetrahedral opposite charge. Shape selectiveness is another notable peculiarity to zeolites owing to their microporous nature. This microporous structure influences development of massive products. Over the course of decades, several mesoporous aluminosilicates (HMS, SBA-15, MCM-41, etc.) have been observed. Mesoporous aluminosilicates possess inadequate hydrothermal firmness and low acidity potency compared to zeolites [29,30,31,32].

Extensive use of solid catalysts in industrial catalytic processes is still challenged by coke deposition. Over the last few decades, scientists have studied this phenomenon extensively, leading to more profound knowledge of coke dynamics in various processes. Catalytic pyrolysis of plastic and biomass heap stocks is considered since it has been shown to be an effective technique for waste revalorisation. Reactivating inactive catalysts became necessary due to economic and environmental considerations. Many regeneration techniques have already been investigated, but the industry still confronts formidable obstacles in creating an effective and long-lasting system. In many industrial processes combining organic chemicals and solid catalysts, such as those in the petrochemical sector, catalyst deactivation is frequently observed. There are a few distinct ways that a heterogeneous catalyst might deteriorate, which ultimately results in the catalyst becoming inactive and losing its catalytic activity. In general, there are five basic mechanisms described in the literature that can cause a catalyst to become inactive: i. poisoning; ii. solid-state processes; iii. mechanical failure of the catalyst; iv. thermal deterioration and sintering; v. fouling, coking, and carbon deposition [33,34]. The deactivation process, known as poisoning, which “blocks” the active catalyst sites by strongly chemisorbing molecules not participating in the reaction, is highly prevalent. This action is performed when the chemisorption of these poisonous species is greater than the affinity of reactants for active sites. Deactivation can also arise when the catalyst reacts unpredictably with species already existing in the process. In order to become inactive, a catalyst may undergo a solid-state reaction or a gas-vapour–solid reaction, both of which involve transition of the catalyst’s surface from an active to an inert phase or into volatile chemicals that escape the reactor as by-products. Additionally, mechanical failure of the catalyst can happen in some circumstances and present itself in several ways, including crushing, attrition, and/or erosion of catalyst pellets, all of which result in essentially irreparable structural damage to the catalyst [33,34].

**Figure 2:**
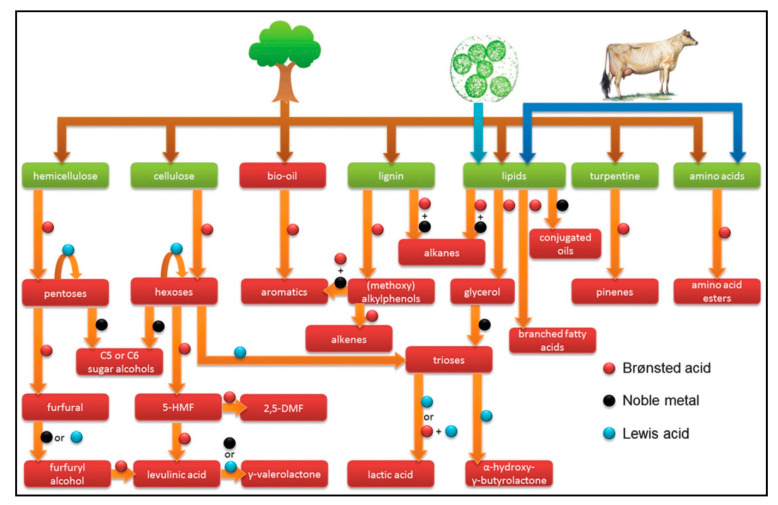
A comprehensive summary of the various biomass conversions and the corresponding catalytic active sites employing zeolites [10] (Open Access).

The fluid catalytic cracking (FCC) process involves splitting the most significant hydrocarbon into gasoline with the aid of zeolites that offer about 45% of the worldwide gasoline reservoir. A large percentage of zeolites possess dehydration, esterification, and acylation methods for catalytic cracking. These zeolites are required for oxygenating (oxygen-containing) compounds’ transformation into hydrocarbons such as oil and modified methanol to gasoline (MTG) production. The biochemical alteration, particularly the fermentation process, transforms carbohydrates into ethanol. Zeolite catalysis involves transition of biomass, such as lignocelluloses and carbohydrates, into transit fuels and chemicals, respectively. The potent acidity in zeolite residuals plays an integral role in oil betterment automation at a broad spectrum in oil refineries [35,36,37,38,39,40,41,42,43]. The different properties of zeolites contribute significantly to conversion of raw materials, including biomass or biomass-derived products. Zeolites have a limited role in the biomass treatment process. However, they play an integral part in several chemical processes that convert biomass to energy and value-added chemicals, as shown in Figure 2 [10]. Zeolites have shown potential performance in fast catalytic pyrolysis (FCP) in platform chemicals, such as glucose/furans or raw biomass feedstocks [44,45,46,47]. The current review considers zeolite catalysts’ role in chemicals and fuel development from biomass material. The review further presents the different types of zeolites and chemistry. Furthermore, the zeolites’ potential and challenges in biomass conversion are critically reported. Finally, the future prospects and challenges associated with zeolites as a catalyst for biomass conversion are elucidated.

## 2. Types of Zeolites and Chemistry

### 2.1. Zeolites Structure, Properties, and Different Forms

**Figure 3:**
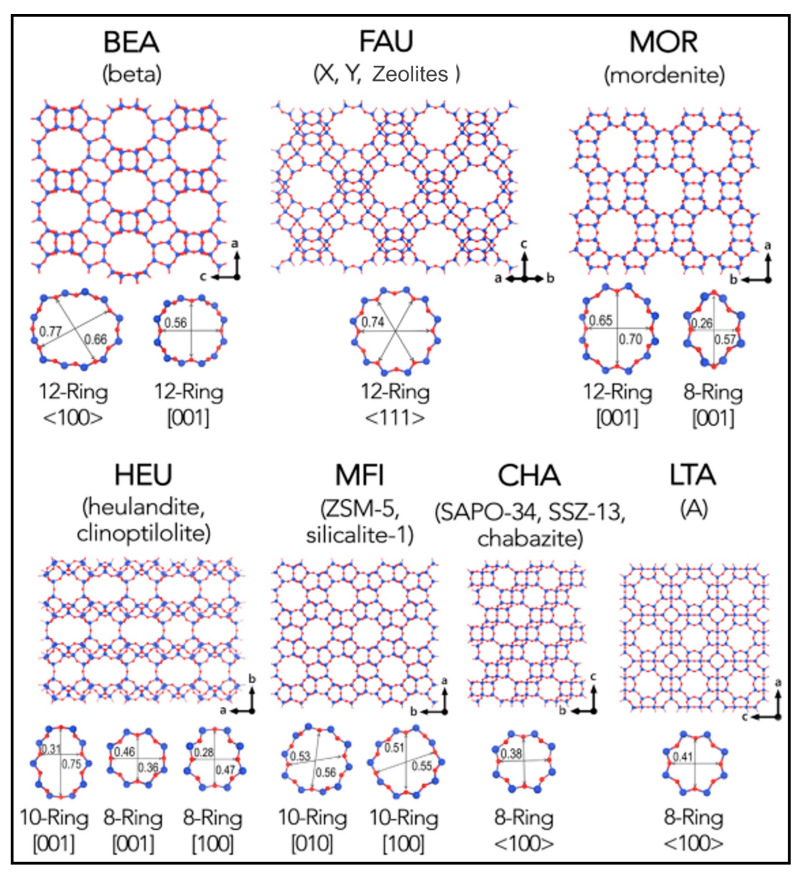
Shows the selected zeolite structures framework assigned with a three-letter code by the International Zeolite Association. Zeolites are classified based on their widest pore windows: small-pore (≤8-ring), medium-pore (10-ring), large-pore (12-ring), and extra-large-pore zeolites (>12-ring) [66].

Wright and Lozinska (2011) stated that zeolites are extremely porous aluminosilicate crystal structures possessing a three-dimensional network [48]. Porous structures aid in capturing molecules as small as 1 nm, resulting in formation of channels or cages. The chemical composition affects the 3D framework. Zeolites undergo chemical transformations throughout the process of production. Charge balancing, organic group incorporation, and oxygen substitution occur due to these modifications [49,50,51,52,53,54,55,56]. Several varieties of zeolites and their chemical structures have been discussed in this research. It pinpoints zeolite properties associated with defects and a highly relevant periodic framework. Król (2020) described the framework for natural and synthetic zeolites alongside their properties and utilisation [57]. Zeolites originating from natural or waste resources are considered in this paper. This paper has provided deep insight into natural and synthetic zeolites. The Zhang et al., 2018 study has demonstrated the molecular filtrate of how mesoporous, y zeolites, hierarchical, and ZSM-5 zeolites are formed [58]. Zeolites’ shape and kind of pore size include massive pore-like Y zeolite, Beta zeolite SSZ-55, and medium pore consisting of SSZ-20, ZSM-23, Ferrierite, MCM-22, TNU-9, IM-5, Small pore comprise of SAPO-34, and ZK-5 that were synthesised, labelled, and examined by GC MS combined pyro probe reactor for the formation of aromatic from the glucose molecule [59,60,61,62,63,64,65]. Figure 3 shows the selected zeolite structures framework assigned with a three-letter code by the International Zeolite Association [66]. Cnudda et al. (2020) study has demonstrated that SAPO-34 zeolites synthesis required the utilisation of TEA (Triethylamine), hydrated alumina, water, phosphoric acid, and silica solution for solution progenitors formation [67].

The pore size of the zeolite catalyst decided the aromatic compound’s gain. The considerable yield of aromatics compounds containing oxygen (arises from glucose pyrolysis), carbon dioxide, carbon monoxide, and coke is not created by small pore zeolites. Average zeolites of 5.2–5.9 Å range pore sizes produce maximum aromatic products. Higher pored zeolites assist in coke making as it includes low oxygenate products, low aromatic products, and high coke yield. Zhang et al. (2019) have stated a strategy to add hydrogen elements to escalate product selection and decrease coke production [68]. This, in turn, enhances catalyst viability with the aid of the catalytic hydrocracking process. Dai et al., 2018 have stated an addition of n- butanol in the MTA process for acquiring the life of the catalyst to 50 h [69].

For aromatic compound formation, internal porosity, window size pore, and steric impediments play an essential role. Steric hindrance (ZSM-11 and ZSM-5) and moderate internal pore space of zeolites possess the smallest quantity of coke and the most significant aromatic compound production. Pharmacokinetic parameters were estimated to identify exterior surface locations or interior locations for diverse zeolite catalysts, reactants, and products. Most aromatic reactants and products are proven to be suitable within the majority of large- and medium-sized pore zeolites. Nevertheless, direct or minor aromatics reactions and secondary reactions on the catalyst’s surface generate polycyclic aromatics compounds. Based on the Taarning et al. (2011) study, different types of zeolites were discussed for advancements in biomass conversion. Standard zeolites were used to convert sugars into valuable chemicals to transform lignocellulose matter fuels and Lewis acidic zeolites [70]. Fossil sources; effective transformation into fuels and chemicals are caused by heterogeneous catalysts. Zeolites have exhibited their potential for biomass transformation in recent times, and it is included under the heterogeneous catalysts category. Wang and Xiao (2015) stated that zeolites’ potent Lewis and Bronsted acidity has made them efficient in the catalytic output of oil refining, petrochemical, and professional chemical products [71]. In addition, attributes such as adsorption capability and quite specific areas possessed by zeolites permit biomass pyrolysis for catalytic reactions, as stated by Resasco et al. (2016) [72]. Ennaert et al. (2016) stated that the hydrophilic and hydrophobic material of zeolites could be replaced, and restrictions on zeolite’s adsorptions features could occur [73]. It has been found that zeolite possesses excellent biomass catalytic conversion ability as compared to other catalysts, as stated by Li et al. (2017) [74].

### 2.2. Mesoporosity, Acidity and Crystal Size Effect

A zeolite-based catalyst’s porosity, acidity, and crystal size all work together to generate appropriate catalytic qualities in terms of activity, selectivity, and stability. These features are provided through the synergistic effect. This research presents several helpful hints and instructions for synthesising appropriate technical zeolite-ZSM-5-based catalyst materials for the catalytic pyrolysis of biomass. These instructions also include analytical methods for evaluating the structure and performance of these catalysts.

For zeolitic materials with weak self-binding capabilities, adding binders is necessary to generate durable catalyst bodies with the appropriate strength and attrition resistance. Generally, these binders are alumina, silica, or clays [75,76,77]. However, the choice of the particular binder or other additives, and even the amounts utilised, are vital since they result in significant changes in the overall characteristics of the catalyst material and activity [78,79,80]. The literature has several illustrations of technological zeolite ZSM-5 based catalysts produced using clay minerals as binders. For example, Pérez-Uriarte et al. conducted research on the conversion of dimethyl ether (DME) into light olefins using zeolite ZSM-5 based catalyst extrudates. Their findings show that agglomeration with boehmite was preferable to extrusion with bentonite in terms of activity and stability [81]. For the methanol-to-hydrocarbons (MTH) reaction, Michels et al. demonstrated that zeolite ZSM-5 was successfully fabricated with attapulgite as clay material [82]. Wang et al. discovered that attapulgite addition improved overall catalytic stability and light-olefin selectivity while attenuating the catalyst’s intrinsic activity. Due to the altered acidity and increased diffusivity, attapulgite-improved zeolite-ZSM-5-oriented catalyst materials strengthen the selectivity and stability of p-xylene in toluene methylation [83,84].

As with several other chemical processes, the choice of catalyst in CFP is crucial for selectively obtaining high-heating value products for prolonged stable durations. Considering its propensity to create a high-quality, highly deoxygenated bio-oil, zeolite ZSM-5 is frequently utilised as a catalyst for CFP of biomass, either alone [85,86] or in conjunction with promoters such as Ni or Ga [87,88]. The superior performance of zeolite-ZSM-5-based catalysts is frequently attributed to their unique pore structure and acidity, which promote the many pyrolysis-related processes, including cracking, deoxygenation, oligomerisation, alkylation, and aromatisation [84,85,89]. Moreover, the zeolite ZSM-5’s low stability is a frequent drawback of this method. In fact, during the pyrolysis event, coke production easily blocks the zeolite ZSM-5 micropores. In biomass conversion processes, introduction of hierarchical ZSM-5 zeolites, which are firmly mesoporous, for instance, as a result of desilication [90], might enhance catalyst stability with time-on-stream [91,92]

In order to generate bio-oil from oak biomass in an ex situ catalytic fast pyrolysis (CFP) process, a number of different technological catalysts based on zeolite ZSM-5 were evaluated and compared in terms of their effect on CFP performance. Microporous ZSM-5-ATP containing attapulgite (ATP as binder) had the lowest activity toward the cracking/deoxygenation process among the catalysts tested. High-tech analysis showed that coke, often formed near strong Bronsted acid sites, concentrates in zeolite-rich domains and is far less present in binder-rich aggregates. With the addition of mesoporosity, which significantly reduces the likelihood of pore blockage, the desilicated variant (i.e., the ds-ZSM-5-ATP catalyst) was more active toward deoxygenation throughout extended reaction durations [93]. By increasing the number of Lewis acid sites, ZrO_2_ inclusion into desilicated ds-ZSM-5-ATP reduced the catalyst’s cracking activity and improved bio-oil output.

The maximum activity in bio-oil deoxygenation was demonstrated by nanocrystalline ZrO_2_/n-ZSM-5-ATP, which generated a high output of organic bio-oil (20 wt % after 25 min of reaction) with the lowest oxygen content (14 wt %). ZrO_2_/n-ZSM-5-ATP, which has a higher surface area and less acidity than microcrystalline catalysts, maintained its acidity and textural characteristics better throughout the reaction [93].

### 2.3. Zeolites Chemistry and Its Design Advancement

**Figure 4:**
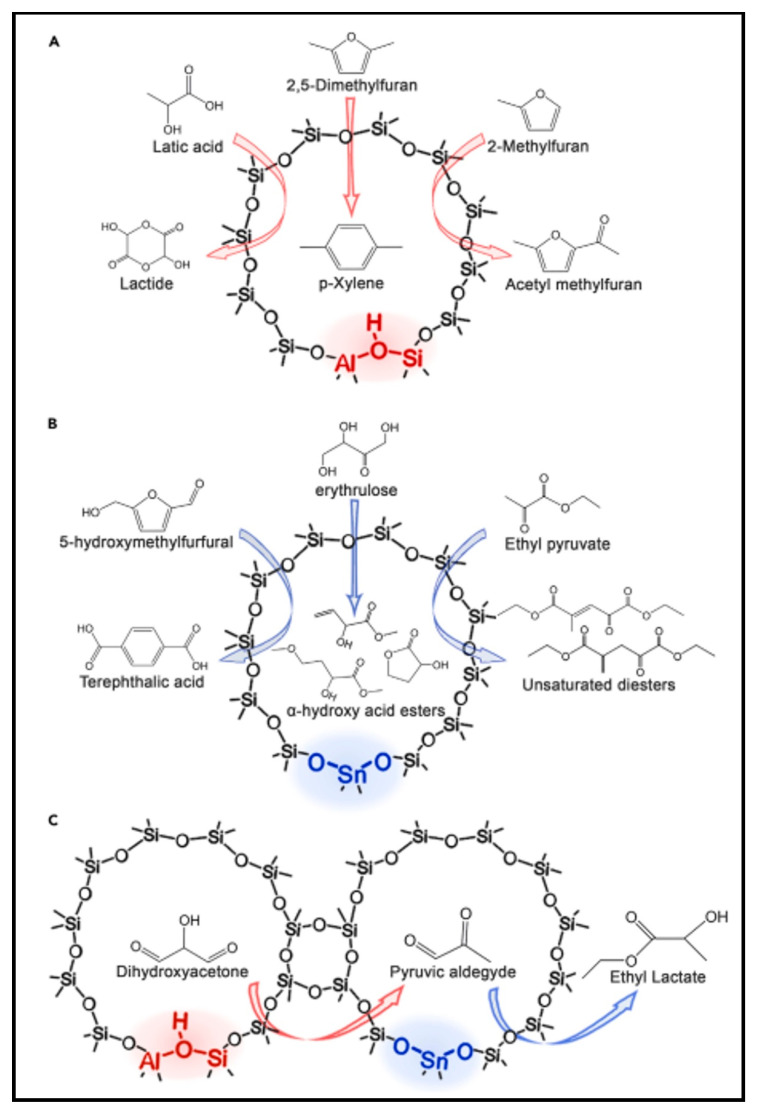
Various pathways of zeolite catalysts for biomass conversion with various active sites: (**A**) Brønsted acid site, (**B**) Lewis acid site, (**C**) multifunctional active sites [66].

Ennaert et al. (2016) stated that conventional refineries and investigation of the substitution of fossil oil with alternate source materials were driven by industry and academics owing to the growing need for fuels and chemicals [73]. Further emphasised was valorisation of medium derivatives and biomass elements with the intent of worthwhile fuels and chemical formation. Therefore, a particular role of zeolite catalysis is being discussed for biomass conversion. It highlighted biomass transformation by the zeolites’ catalysis process and its association with prevailing petrochemical procedures. Designing prime physicochemical characteristics for zeolites is important. Due to these features, zeolites have acquired the potential to expand the range of biomass conversion approaches. In connection to hydrodeoxygenation, procedures include hydrotreatments, pyrolysis, catalytic cracking, a considerable amount of condensation, dehydration, and isomerisation reactions. Therefore, advancement in categorised zeolites reasonably demonstrated the likelihood of introduction, strengthening the availability of a diverse variety of active sites (metallic stages, primary sites, Bronsted, and Lewis acid centres). Figure 4 shows zeolite catalysts chosen to illustrate biomass conversion in various active sites. The outstanding performance of zeolites in a variety of biomass conversion routes is based on these characteristics [66].

Although it is usually recognised that Bronsted acid zeolites are effective for cracking reactions, it is still difficult to pinpoint the locations of acid sites and manage their dispersion. Research has demonstrated that the Si/Al ratio and the synthesising process seem to impact the distribution of Al atoms in a zeolite, which could also result in variable catalytic activity for the same kind of zeolite framework [94,95]. Because of this, attempting to compare experimental results from different groups that used zeolites from various sources requires caution. Janda and Bell have looked at how the Si/Al ratio affects the distribution of framework Al and how that affects butane cracking and dehydrogenation in H-MFI [96]. While the Al concentration rises, they discovered that the Bronsted protons tend to be placed around channel junctions. Dehydrogenation prefers to originate at channel contacts over cracking reactions because the transition state geometry for dehydrogenation is bulkier and demands larger space than for cracking reactions. Therefore, altering the Si/Al ratio of the zeolite might alter the output selectivity of alkane cracking and dehydration. The positions of the protons can also impact the characteristics of the acid in addition to the confinement effects [97].

Resasco et al. (2016) stated that maintenance of the greatest acceptable carbon amount from C7 to C15 in fuel involves removal of oxygenated functional groups in addition to carbon–carbon bond generation for fuel production from biomass [72]. Elements constituting oxygenated functionalities, such as hydroxide, carboxylic, and carbonyl groups, are employed for catalysing C–C bond-forming reactions, such as alkylation, ketonisation, and acylation, with the aid of acidic zeolites. In addition, feasible mechanisms of these reactions and the nature of active sites are also being elaborated. Fu et al. (2020) stated that the ZSM-5 zeolites possess a three-dimensional network system of pore size (5.2–5.5 Å) [98]. Restriction on adverse and competitive reactions occurs with the aid of ZSM-5 owing to its microporous nature. Zeolites provide a tremendous contribution to reactions, such as alkylation, cyclisation, and aromatisation, as stated by Hu et al. (2020) and Kumar et al. (2020) [99,100]. Guefrachi et al. (2020) elaborated that zeolites’ active centre contains acidic sites. It has been found that acidic site strength decides application specificity, as stated by Xin et al. (2019) [101,102,103]. Peng et al. (2020) stated that potent adsorption ability and higher surface area are possessed by zeolite molecular sieves [104]. Reduction in oxygen levels is required when using zeolites to enhance bio-oil products. Low-value bio-oils are converted into high-value chemicals as a consequence of this process. The Shamzhy et al. (2019) showcased zeolite structure contains multiple Al^3+^ and O_4_ molecules that help counter the average negative charge on zeolites [105].

Current revelations in computations using density functional theory (DFT) also help to explain the precise chemistry of catalytic processes in zeolites. Perhaps the periodic or cluster models can be used to conduct these computations. Although systematic DFT simulations for zeolite systems can capture interactions from extended framework structures, they are frequently computationally intensive due to the requirement of large unit cells [106]. The unphysical border phenomenon may result in inconsistent results when utilising small cluster models, which can significantly cut processing costs. The hybrid quantum mechanics/molecular mechanics (QM/MM) technique, which only uses quantum mechanics to interpret a tiny cluster enclosing the active core and a classical force field to explain the rest of the zeolite framework, has been used in published findings to overcome these problems. Despite significantly raising computing expenses, this hybrid technique can accommodate long-range van der Waals and electrostatic interactions in porous environments [106,107]. To determine precise enthalpies and entropies, low-frequency modes corresponding to translational and rotational motions between alkanes and zeolites should always be handled appropriately. Due to their potential energy surfaces’ typical high anharmonicity, these low-frequency motions of weakly coupled molecules are notoriously challenging to model [108]. Because of this, notable researchers have incorporated a variety of computational techniques, including the mobile block Hessian method (MBH) [109], quasi-rigid rotor harmonic oscillator (quasi-RRHO) [110], and configurational-bias Monte Carlo (CBMC) [111], to more accurately model the adsorption and activation of alkanes in zeolites.

### 2.4. Carbohydrates Chemical Science

**Figure 5:**
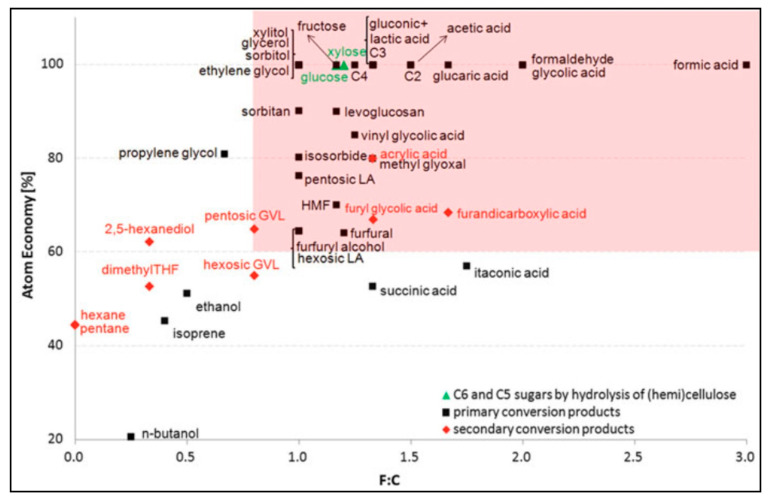
Graphical representation of the atom economy vs. F:C for common carbohydrates and their derivatives. Graph and data are reproduced with the permission of Springer (based on Dusselier et al., 2014, pp. 1–40) [112].

A potential approach for generating sustainable energy is conversion of woody biomass into biofuels. Consolidation of biorefineries leads to a low-profit margin technique but extensive production volume. The manufacturing procedure deserves to be economical to contend with petrochemistry refineries. Biorefinery is expected to generate top-quality synthetics adjacent to the low-functionalised biofuels. Due to plentiful chemical functionalities in carbohydrates, the hemicellulose sugar portion of biomass is sufficient to form top-quality chemicals. A prudent option for goal-oriented molecules has become imperative as hemicellulose polysaccharides create various chemicals. The Dusselier and Sels (2014) study has estimated the top chemical prospects in biorefinery for carbohydrates [112]. The functionality index (F:C) evaluates functional groups and their amount standardised to carbon atoms. Calculation of atom efficiency/percentage associated with principal derivatives and products of biomass by using F:C against corresponding polysaccharides or monosaccharides was shown in Figure 5.

Low F:C was observed in petroleum derivative molecules, such as alkanols, alkenes, and alkanes. High F:C group is defined by product and biomass elements, as shown in Figure 5. F:C value is contingent upon a given chemical. When there is huge demand, a small F:C value of the molecule can be worthwhile. The majority of the high-interest elements possess diverse chemical transformation tracks. Five distinct reactions are isomerisation, retro-aldol, dehydration/rehydration, hydrogenation, and oxidation.

## 3. Potential and Challenges of Zeolite

### 3.1. Zeolites Role in Environment Protection

Rhodes (2010) stated that zeolites are aluminosilicate solids that possess a negative charge on the micropores’ honeycomb structure [113]. This structure adsorbs several molecules to remove environmental contaminants with the intent of catalysing chemical reactions. Due to organic solvents’ lesser requirement, zeolites are a key part of sustainable chemistry. For smashing crude petroleum portions into chemical source material and fuels for commercial operations, proton ion-exchanged zeolites are broadly involved in the oil industry. Zeolites are worthwhile at the commercial level for water softening by expelling radioactive cations from nuclear decay and eliminating noxious metal cations from underground and surface water. SMZ (surfactant-modified zeolites) received special consideration for extracting poisonous anions and organic contaminants. Through adsorption, zeolites eliminate hazardous anions, such as chromate, arsenate, radioactive iodide, and arsenite since zeolites are packed with Pb and Ag cations. As a result, zeolites form insoluble aggregates inside their matrix.

### 3.2. Zeolites for Biomass Transformation into Fuel, Chemical Feedstock, Aromatics, and Levulinic Acid through Several Processes

Bahri and Anugra (2011) stated that the need for global crude oil rises during a stock shortage [114]. Figuring out renewable assets is inescapable owing to fossil fuel restrictions in the upcoming future. Biomass pyrolysis creates bio-oil, which is a substituted energy source. To convert biomass into fuel, pyrolysis is a viable option that results in bio-oil from biomass heating without oxygen. Oil palm solid trash fibre, trunk, and shell have all been utilised as specimens. In the pyrolysis reactor, silinap 500 mL, 50-g biomass, and Ni/NZA catalyst of 1.5 g at 320 °C were employed for catalytic pyrolysis procedures. In triggered natural zeolite (NZA), consequences of nickel–metal ratio in catalysts of 1, 3, and 5 wt/wt % were investigated. For trunk possessing a particle size of −8 + 10 network with the Ni catalyst of 3%, the utmost bio-oil of 63.0% is achieved. The bio-oil outcome is examined, including viscosity, acidity, density, and flashpoint. GC–MS was utilised to assess bio-oil chemical elements

**Figure 6:**
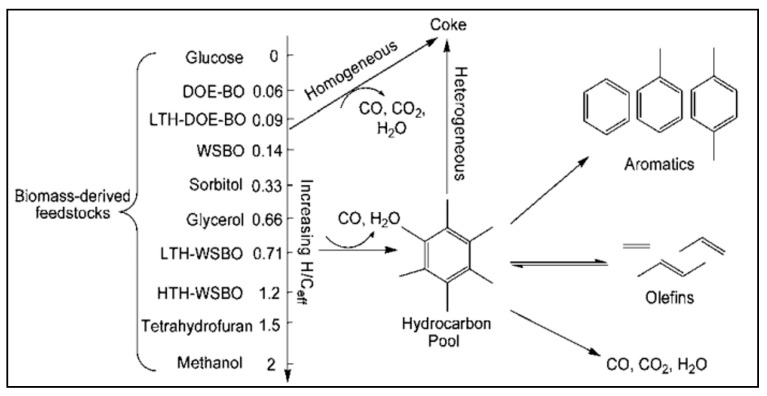
Reaction schematic of biomass-derived feedstocks with ZSM-5 catalyst (Zhang et al., 2011, The Royal Society of Chemistry) [116].

Goshima et al. (2014) stated that hydrothermal reactions of fermenting molasses or bagasse demonstrated the catalytic transformation of oxygen possessing biomass intermediates, such as butyric acid, acetic acid, levulinic acid, and furfural. Zeolites such as SAPO-34, SAPO-11, and ZSM-5 were examined to convert biomass derivatives propylene and BTX [115]. For generation of BTX with the aid of ZSM-5, acetic acid and levulinic acid were found to be appropriate. However, the butyric acid was worthwhile for chemical feedstock formation with the help of the ZSM-5 catalyst. Butyric acid conversion into propylene at good yields occurred through SAPO-11 catalyst. The output for the propylene molecule was found to be an extreme value of 58.8 C% at 723 k. The propylene to gaseous hydrocarbon products ratio was enhanced to 90.4 C%. Furthermore, schematic reactions of biomass-derived starting material with the assistance of ZSM-5 catalyst are represented in Figure 6.

According to Stefanidis et al. (2019), the CFP (catalytic fast pyrolysis) process transforms lignocellulose biomass into a deoxygenated liquid power source, such as bio-oil and precious chemicals, which has distinct properties when compared to the conventional quick pyrolysis process for bio-oil development [117]. Biomass thermal decomposition reaction on heterogeneous catalyst surface creates deoxygenated vapours and breaks for suitable yield formation. It has been studied that ZSM-5 zeolite is one of the most frequently investigated candidate catalysts for CFP. The Jae et al. (2011) study showed ZSM-5 as an efficient catalyst owing to its unusual micropore shape and acidity [118]. This structure is highly shape-selective to reduce undesirable coke and the single aromatic hydrocarbon formation. Still, the thermal decomposition of biomass forms oligomers and massive oxygenates, which react on the catalyst’s restricted external surface area and cannot disperse into the catalyst ZSM-5 micropores. The growing relevance of hierarchical mesoporous zeolites results in them being candidate catalysts for the procedures since ZSM-5 catalyst of an entirely micropore framework might not be optimum for the CFP biomass. Catalyst materials integrate a high level of acidity, with zeolites shape selectivity facilitated by a secondary mesoporous system that enhances accessibility.

Diverse groups have examined that hierarchical zeolites synthesis occurs through the desilication method for CFP biomass in miniature scale reactors, as stated by Gamliel et al. (2016); Shao et al. (2016); Ding et al. (2017) [119,120,121]. Li et al., 2018 presented the treatment of desilication by adulterating La and sodium carbonate [122,123]. Zeolite’s materials performance is better in the context of suitable yield production and activity when the desilication process is conducted under light conditions. The materials’ performance worsens under extreme desilication situations, most probably due to macropore generation, shape-selectiveness, severe absence of microporosity, and decline in zeolite structure.

Deng et al. (2014) stated that biofuels and valuable chemicals generation from natural renewable sources like biomass. In the study, shavings conversion into aromatics compounds like xylenes, toluene, and benzene with the aid of HZSM-5, NaZSM-5, HY, and ReY zeolite catalysts [124]. HZSM-5 catalyst is one of the examined catalysts that show the aromatics compound generation containing utmost activity. HZSM-5 also possesses water impermeability, suitable acidic features, catalytic decomposition, isomerisation, and impressive selection with the intent to form aromatic compounds, as stated by Kim et al., 2017 [125]. Jia et al., 2017 have showcased hierarchical zeolite preparation from HZSM-5 Zeolites by NaOH removal [121,126]. Liao et al., 2020 have constructed a ZI40 Hierarchical catalyst [127].

**Figure 7:**
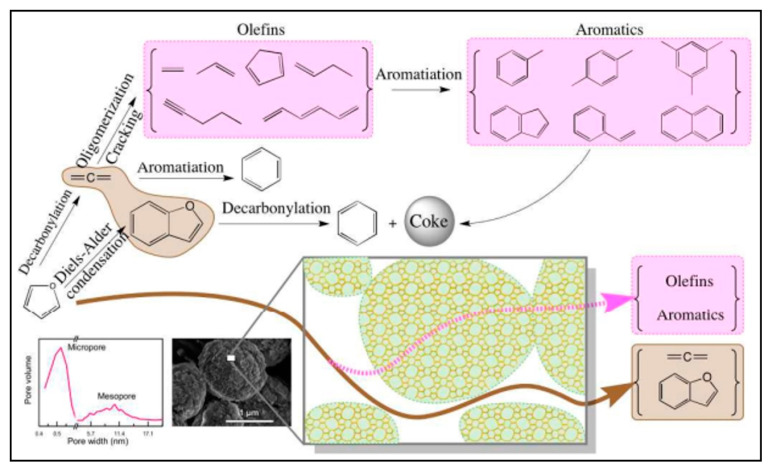
Furan conversion with the assistance of meso ZSM-5 catalyst (Gou et al., 2017, copyright 2014, The Royal Society of Chemistry) [129].

Aromatic compounds’ carbon selectiveness and product yield are about 62.5 degrees C and 26.5% mol%, respectively, under the ideal circumstances of catalyst/lignin ratio of 2, f(N_2_) = 300 cm^3^/min and T = 450 degree C owing to HZSM-5 catalyst. The effect of reaction parameters, such as catalyst/sawdust ratio, gas flow rate, and temperature, on the synthesis of aromatic compounds was investigated, as well as transformation of lignocellulose biomass into aromatics. Uslamin et al., 2019 showcased hierarchical ZSM-5 zeolites-oriented reactions using methanol from furan compound [128]. As shown in Figure 7, several conversions linked to aromatic compound formation are found.

**Figure 8:**
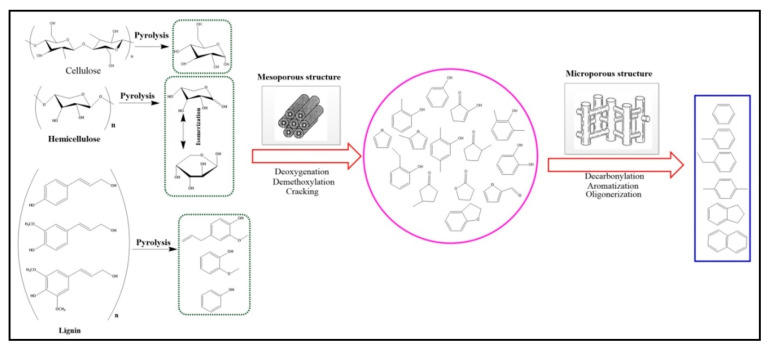
Lignocellulosic reaction path with the help of hierarchical zeolites (Li et al., 2020, Elsevier) [130].

Comprehensive estimation was executed by employing several assessments, such as FTIR, SEM, XRD, AAS, BET, and EDX zeolite catalysts. Evaluation has demonstrated that primarily mesoporous ZSM-5 or hierarchical catalyst has included characteristics compared to the microporous equivalents. Formation of dignified rice husk reactions to levulinic acid (platform chemicals) required a catalyst. In a similar reaction, a certain proportion of MnCl_2_.4H_2_O was utilised as a homogeneous catalyst. HPLC technique was employed to analyse the isolated yield of reactions. By hierarchical zeolites MnOx/hi_ZSM-5 and microporous MnOx/mi_ZSM-5 catalysts, levulinic acid was generated at 15.83% and 10%, respectively, under appropriate conditions of eight hours for transformation, and homogeneous Mn (II) catalyst endorses creating 8.86% levulinic acid yield possessing additional charcoal as a product. In addition, by assessing EDX records and FTIR spectra, the constancy of the zeolite catalysts after the reaction (isolated and calcinated at 550 °C) was explored. Analysis reveals reactions showing manganese oxide separation out of structure due to the acidic condition and alumina and silica. However, biotransformation results in an interplay between Mn^2+^ ions (solution) or MnOx in zeolites and cellulose with a porous ZSM-5 framework. The hierarchical system assists the ZSM-5 structure of the catalyst to remain integrated. Li et al., 2020 discussed that aromatic hydrocarbon synthesis via microwave-aided catalytic fast pyrolysis (MACFP) of rice husk was studied using alkali-treated HZSM-5 zeolite (Figure 8) [130].

Narula et al. (2015) stated that the hydrocarbon mixture stockpile generates from ethanol through the direct catalysis process [131]. It has been observed that a 10–15% ethanol mixture can result in rising in biofuel utilisation in modern automobiles. Zeolite-catalysts-linked transformation of ethanol has been elaborated on via literature studies, but this strategy is unable to meet commercial expectations owing to high hydrocarbon content. Additionally, it has been studied that the endothermic dehydration stage is involved in ethanolic conversion based on earlier mechanistic research. The study has announced that, in V-ZSM-5, the catalyst forms hydrocarbons from ethanol without hydrogen addition. This catalyst creates <13% carbon content compared with the H-ZSM-5 catalyst. In situ DRIFT and C_2_H_5_OD experimentation elaborated that the majority of the yields from the dehydration stage and hydrocarbon reservoir mechanisms are not significant enough. Several paths have been discussed for direct ethanolic conversion to a suitable hydrocarbon mixture. The hydrocarbon blend mixture creates fuels such as JP-8, gasoline, jet fuel, diesel, and BTX, commodity chemicals at the refinery. Hasan and Jazie (2021) stated that biomass conversion into high-value fuel for misapplication is the most notorious practical path [132]. Among the most effective strategies for bio-crude oil development from damp biomass is hydrothermal liquefaction. However, there is a requirement for expensive up-gradation remedies, such as biofuel.

It is essential to utilise a catalyst to improve the bio-crude product. It strengthens bio-crude products using heterogeneous catalysts. Various catalysts can be distributed into four noticeable categories: alkaline metal oxides, lanthanides oxides, transition metals, and zeolites. Due to the conventional catalytic activity of lignocellulosic biomass by hydrothermal liquefaction process, bio-crude level and quality are affected due to catalysts’ hydrodeoxygenation action. A maximum output of bio-crude usages has affirmed lanthanide oxides and metals are shifted. Simultaneously, it can offer high-production bio-crude quality assurance. This investigation’s objective is to outline the effect of zeolite catalysts’ addition on lignocellulosic biomass hydrothermal liquefaction with the intent to facilitate biocrude effectiveness and yield. The study has also emphasised the impact of catalysis equivalent to zeolite catalysts [133,134,135,136,137,138,139].

### 3.3. Challenges Associated with Zeolites Structure

Luo et al., 2016 demonstrated that oxygenated elements’ activity and transformation require the necessity of zeolites possessing heteroatom, such as Ta, Hf, Zr, Sn, and Nb, because of tetrahedral metallic sites with unusual Lewis acid features [15]. Hydrophobic Lewis acid zeolites are formed by fluoride-linked synthesis that serves as water-tolerant catalysts. Many unanswered questions surround the properties of these zeolites, which are especially concerning in terms of active site characteristics. Numerous discordant findings in the current literature records were illustrated by inadequate basic knowledge. The study employed the molecular-based strategy to connect zeolites’ metallic-centric framework and reactiveness for various substrates to acquire a robust structure. Soltanian et al. (2020) described lignocellulose biomass transformation through CFP by devising extremely active catalysts [140]. The deoxygenation rate of pyrolysis vapours or gases is facilitated by sophisticated catalysts that help escalate fuel precursors generation. Conducive settings for catalytic pyrolysis vapours into transit fuel value type are conducted by zeolites. However, the nano-scale framework restricts reactants’ diffusion into hyperactive locations and apertures. Exterior micropore significant elements agglomeration obstructs pore channels and enormous coke development. Thus, it results in prohibition of acidic sites availability. Due to this reason, the catalyst’s unsteadiness and rapid inactivation occur, which give rise to catalyst restoration and rehabilitation.

An encouraging field is associated with hierarchical zeolites of a micro- or mesopore framework to deal with the indicated opposition. In zeolites, the availability of mesoporosity production and acidic sites and a customised pore framework offers sufficient space and supplementary dynamic stages deposit, such as metallic nanometre-scaled particles, strengthening the catalytic activeness. Several strategies have been utilised for modifying zeolites that possess both pros and cons, as shown in Table 1.

## 4. Future Prospects and Recommendations

Zeolite chemistry plays a tremendous role in the fundamental and applied type of investigation. Zeolites act as principal substrates due to development of innovative techniques in the field of physical and computerised chemistry [142]. Thus, magnificent propagation concerning the amount and category of molecular sieves has been identified through investigation, which magnifies the likelihood of innovative paths in catalysis and selective segregation for advancement in zeolites. Rabo (2002) has mentioned the future prospects in zeolite science [143]. Catalytic action, firm acidity linked with multivalent cations, and protic-formed Y zeolites have been investigated. However, new-generation catalysts with steadiness in catalytic sites have not been obtained yet. The underlying cause that fails to acquire uniformity in acidic sites depends upon chemical reactiveness associated with zeolites’ tetrahedral structure from aluminium ions to water, unsteadiness, and flaws in the structure that are created by aluminium hydrolysis under steam. Uniformity in catalytic sites is suitable for all catalytic usages, aiming to eradicate catalytically non-selective and hyperactive sites. The stereospecific catalysis process requires steadiness in catalyst sites. Therefore, the urge for stereospecificity is significant enough in therapeutic use. Therefore, it is vital to emphasise future zeolites of uniform active sites.

Furthermore, advancement in zeolite classification, synthesis, and catalytic action or specificity has been observed. In the future, this leads toward major objectives associated with heterogeneous catalysis, with the intent to make solid crystals of active sites containing uniformity. This opens up a wide range of possibilities in applications using the catalytic process. Zeolites possess catalytic sites of a uniform nature that are obstructed through the synthesis process. Therefore, investigation in this area will be significant enough in the future when zeolite catalysts’ stability at chemical and thermal levels is adequate for use in industries.

Several obstacles associated with catalytic transformation should be considered in the forthcoming years, such as: (i) interplay metal adulteration, acidity, and pores in zeolites lead to enormous progress in product selection; (ii) comprehension of zeolites-linked pyrolysis mechanism for biomass transformation; (iii) exploration of pre-treatment and ultrasonic treatment for zeolites-linked catalytic pyrolysis stated by Luo et al. (2014) [15,144], and in addition, alteration in MFI zeolites remains to be explored as per Palizdar et al. (2020) [145]; (iv) alteration in template and framework guided agents with the intent to construct ranked micro- or mesoporous zeolites; (v) assessment of mass production viability and financial rewards for biomass utilisation progress with the aid of the pyrolysis method.

## 5. Conclusive Remarks

In recent years, utilisation of zeolites in biomass conversion processes has advanced dramatically. More precise biomass component conversion technologies progressively replace traditional biomass cracking processes, comparable to today’s oil-based petrochemical industrial activities. Consequently, lignocellulosic biomass can be transformed into sustainable feedstock to produce bulk chemicals and fuels in the coming years. Zeolites have the potential to be used in this sort of process, and their availability and tunability make them a fascinating prospect to investigate. Several new approaches to selectively producing product line molecules have been discovered in recent years and are currently being studied in great detail. While this is critical for developing renewable bulk chemicals that may be utilised in various applications, it is also essential for reducing the chemical industry’s overall environmental impact.

In response to rising demand for fossil fuels, rising manufacturing prices, and growing environmental concerns, valorisation of cheap, widely accessible, and renewable biomass waste is highly beneficial for industrial applications using zeolite-based catalysis. The amount and variety of biomass waste accessible today offer the opportunity to create useful chemical frameworks and valuable goods. These goods deserve restoration since they may be used in various industrial sectors, such as the chemical, pharmaceutical, cosmetic, and food sectors.

Furthermore, significant progress has been made regarding the importance of structures and reacting functional groups in the effectiveness of zeolites’ catalytic process. It is still debatable whether confinement space affects zeolites’ essential activity and selectivity, particularly in the presence of solvents, and how best to interpret them. Using theoretical research on this subject might be a valuable tool in developing novel catalytic systems in the future. To make effective use of zeolites for biomass conversion into value-added chemicals, it is required to have a thorough understanding of the chemistry of zeolites. Moreover, exploring zeolite’s potential and the challenges associated with biotransformation are vital. Therefore, future prospects and recommendations have been suggested for zeolite’s catalytic transformation.

## Figures and Tables

**Table 1:** Effects of modified zeolites [141].

Modification	Advantages	Drawbacks
Top-down approach	Micropores count and volume must be increased.	The zeolite structure is destroyed.
Bottom-up approach	Boost the zeolite’s mesoporosity and mass transfer efficiency	Hard to pick a template.
Metal doping	Introduces Lewis acid sites for adjusting the acid ratio	The surface area and pore volume of the BET are reduced.

## Data Availability

All relevant data are included in the paper.
